# Comparison of the clinical performance of the flexible laryngeal mask airway in pediatric patients under general anesthesia with or without a muscle relaxant: study protocol for a randomized controlled trial

**DOI:** 10.1186/s13063-018-3141-2

**Published:** 2019-01-09

**Authors:** Sung Hye Byun, Soo Jin Kim, Eugene Kim

**Affiliations:** 1 Department of Anesthesiology and Pain Medicine, Daegu Catholic University Hospital, School of Medicine, Daegu Catholic University, Daegu, Republic of Korea; 2 Department of Anesthesiology and Pain Medicine, Daegu Catholic University Hospital, Daegu, Republic of Korea; 3 Present Address: Department of Anesthesiology and Pain Medicine, College of Medicine, Hanyang University, 222 Wangsimni-ro, Seongdong-gu, Seoul, 04763 Republic of Korea

**Keywords:** Rocuronium, Muscle relaxant, Flexible laryngeal mask airway, Pediatric airway, Oropharyngeal leak pressure

## Abstract

**Background:**

The insertion of a laryngeal mask airway (LMA) is difficult in children due to the unique features of their airways. Muscle relaxants have been reported to facilitate LMA insertion in adults; however, there is a lack of evidence supporting this in children. This trial is designed to assess the feasibility of LMA insertion with and without the use of muscle relaxants in pediatric patients under general anesthesia.

**Methods/design:**

This is a prospective, double-blind, single-center, parallel-arm, non-inferiority, randomized controlled trial to be conducted with participants aged 2–7 years who are undergoing elective ophthalmic surgery under general anesthesia. Participants are randomly assigned to one of two groups based on whether muscle relaxants are used (MR group, *n* = 64) or not used (Saline group, *n* = 64) prior to LMA insertion. The primary outcome is the oropharyngeal leak pressure (OLP) at a fixed gas flow of 3 L/min. The secondary outcomes are intubation time for successful insertion, ease of insertion and mask bagging, intubation attempt for successful insertion, successful insertion rate on the first attempt, fiberoptic view of the LMA position, postoperative complications, and changes in hemodynamic and ventilatory parameters.

**Discussion:**

We will compare the OLPs to determine whether the muscle relaxant provides better conditions for the manipulation of the LMA. This is the first randomized controlled trial to investigate whether muscle relaxants are beneficial to the clinical performance of LMA insertion in pediatric patients under general anesthesia. This trial will be a resource for improving the process and safety of pediatric LMA insertion under general anesthesia.

**Trial registration:**

ClinicalTrials.gov, NCT03487003. Registered on 18 April 2018.

**Electronic supplementary material:**

The online version of this article (10.1186/s13063-018-3141-2) contains supplementary material, which is available to authorized users.

## Background

The use of a laryngeal mask airway (LMA) is increasing in pediatric anesthesia, because it provides less direct mechanical stimulation of the airway since it is placed above the larynx. Several studies have compared the insertion of an LMA and a tracheal tube and showed a significantly increased incidence of perioperative respiratory complications when using the tracheal tube in children [1–3]. However, LMA insertion can be more difficult in children than in adults. The unique features of the pediatric airway, including a larger tongue, a larger and floppier epiglottis, a more cephalad and anteriorly located  larynx, a more acute angle of the posterior pharyngeal wall to the floor of the mouth than in adults, and tonsillar hypertrophy may interfere with the ideal positioning of an LMA [4–7].

Neuromuscular blocking agents, also called muscle relaxants, have long been used to facilitate the insertion of airway devices. There are pros and cons, however, for the efficacy of muscle relaxants in LMA insertion. Recently, several studies have reported that muscle relaxants may facilitate the insertion of LMAs by enabling higher successful insertion rates, higher sealing pressure, lower leakage volume, and lower difficulty of insertion in anesthetized adult patients [8–10]. Conversely, Chen and colleagues showed that sealing pressure, airway pressure, insertion rates, and sore throats did not differ based on muscle relaxant administration [11]. However, these studies were all conducted in adult populations, and there has been a lack of evidence as to whether the muscle relaxants can be helpful in facilitating the insertion of an LMA in children. Therefore, we designed this randomized controlled study to test the hypothesis that the ease of insertion of an LMA would not be compromised in children without muscle relaxants compared to those given muscle relaxants prior to insertion.

## Methods

### Study design

**Fig. 1:**
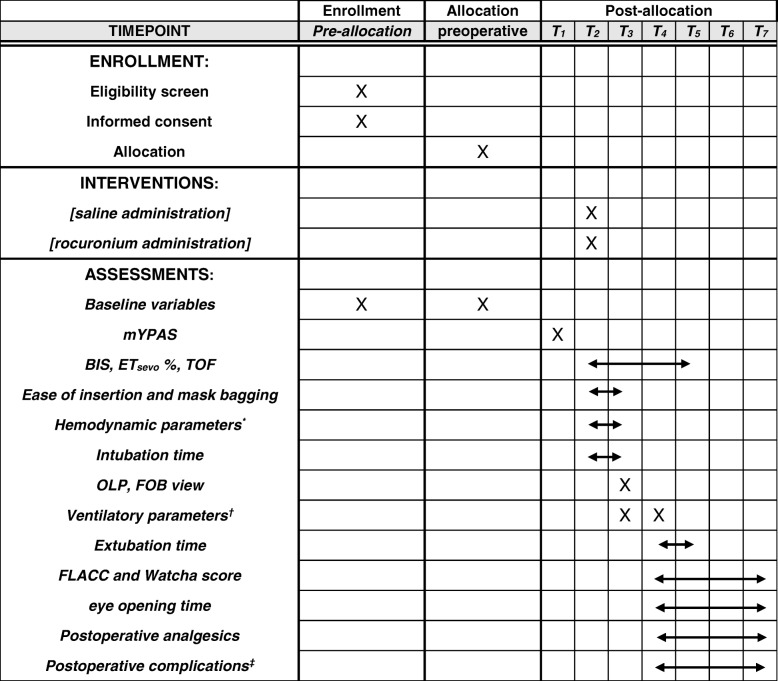
SPIRIT flow diagram: schedule of enrollment, interventions, and assessments. *Including mean blood pressure and heart rate, ^†^ Including ventilator parameter changes such as peak inspiratory pressure (PIP) and expiratory tidal volume to set tidal volume ratio (TVe/TVs) before and at the end of surgery, ‡including respiratory adverse events (coughing, laryngospasm, bronchospasm, postoperative stridor, and desaturation; SpO2 <95%), gastric insufflation, excessive secretion, postoperative nausea and vomiting, sore throat and bloody tinge on the LMA surface; T_1_ at preoperative waiting area, T_2_ after the loss of consciousness is achieved, T_3_ insertion of LMA, T_4_ cessation of anesthetics, T_5_ extubation, T_6_ PACU arrival, T_7_ PACU discharge. *mYPAS* modified Yale Preoperative Anxiety Scale, *BIS* bispectral index, *ET*_sevo_ % end-tidal sevoflurane concentration, *TOF* train of four, *OLP* oropharyngeal leak pressure, *FOB* fiberoptic bronchoscopy, *FLACC* Face, Legs, Activity, Cry, and Consolability

This prospective, single-center, parallel-arm, double-blind, randomized, non-inferiority trial was approved by the Institutional Review Board (IRB) of Daegu Catholic University Medical Center (version 1.2, reference number CR-18-027, validated on 18 April 2018) and was registered at ClinicalTrials.gov (NCT03487003) before patient recruitment. This trial is conducted in a tertiary university hospital (Daegu Catholic University Medical Center) in South Korea. We will conduct the protocol of this trial according to the Consolidated Standards of Reporting Trials (CONSORT) statement. We will follow the Standard Protocol Items: Recommendations for Interventional Trials (SPIRIT) guidelines and flow chart (Fig. 1 and Additional file 1).

### Participants and recruitment

Pediatric patients aged 2–7 years with an American Society of Anesthesiologists physical status (ASA PS) of 1 or 2 who are planning to undergo ophthalmic surgery under general anesthesia are enrolled. The exclusion criteria are as follows:

- Current upper respiratory infections or other respiratory symptoms
- Oral or facial anomaly
- Poor dental condition
- Those who cannot open their mouth or with limited ability to open their mouth
- When tracheal intubation is required

Eligibility is assessed by staff anesthesiologists and/or residents of our hospital. Potential participants who meet the inclusion/exclusion criteria are recruited at outpatient clinics or during preoperative visits before surgery. Since we enroll minors, we provide verbal and written information to, and obtain written informed consent from, all the legal guardians of the participants prior to any study-related procedures (SJK).

### Randomization and blinding

**Fig. 2:**
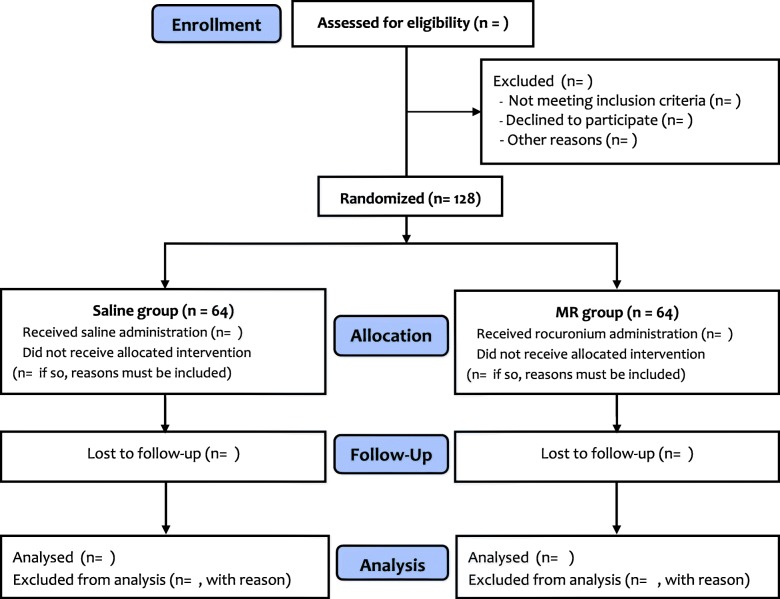
CONSORT flow chart

After recruitment, the participants are randomized to either the Saline or the MR group with a 1:1 ratio (Fig. 2) using a computer-generated random number. The random sequence is managed and kept within sealed opaque envelopes by an anesthesiologist (JHK) who is not listed in the author list (see “Acknowledgements”). When a patient is enrolled in the study, an anesthesia nurse opens a sealed envelope and prepares the study drug according to the group designation. The study drug (rocuronium 0.3 mg/kg in the MR group and saline 0.3 mg/kg in the Saline group) is prepared in a 3-cc syringe and labeled with a white sticker stating “study drug.”

Participant group designations are concealed to all investigators and participants except the anesthesia nurse who delivers the study drugs to the investigator. If serious adverse events that threaten the safety of patients occur (such as death or irreversible injury), we will stop the intervention immediately, break the blinding, and contact the IRB. Intraoperative data recording is done primarily by one of the investigators who insert the LMAs (EK or SHB), and they are not allowed to view the random code. After the post-anesthesia care unit (PACU) admission, one of the two pretrained anesthesia nurse (YSL or YJJ) who does not know the group allocation will measure agitation and pain scores.

### Withdrawal, dropout, and discontinuation

Participants are free to withdraw at any time during the trial at their own or their legal representative’s request. If the continuation of the trial may threaten the participant’s health, a participant can be withdrawn based on the investigators’ judgement. Reasons for withdrawal are recorded on a case report form. All data are analyzed according to the intention-to-treat principle.

### Confidentiality

Personal information including names, social security numbers, or chart numbers will not be collected. Personal data such as sex, age, weight, height, and the date of operation (but not names, social security numbers, or chart numbers) are recorded on a separate Excel file and kept secret until the end of the study. This Excel file will be accessible only to JHK, but he is not allowed to access the collected study data. Finally, personal data and the study code will be combined and analyzed after the end of the study, and this dataset will be managed by the chief investigator (EK). After the completion of the study, the collected data will be encrypted and stored for 3 years and then discarded.

### Intervention

In the preoperative waiting room, patients are premedicated with midazolam (1–1.5 mg/kg) intravenously in the presence of the parents. When a patient enters the operating room, standard intraoperative monitoring (non-invasive arterial blood pressure, electrocardiography, and pulse oximetry), bispectral index (BIS; VISTATM monitoring system, Aspect Medical Systems Inc., Norwood, MA, USA) and acceleromyography (TOF-watch®, Organon Ltd., Dublin, Ireland) are applied. Anesthesia is induced with 6–8 vol% sevoflurane under 100% O_2_ mask ventilation. When the patient is sufficiently unconscious (BIS under 60, showing no responses to jaw thrust and loss of eyelash reflex), the study drug is administered. Saline 0.3 mg/kg is given in the non-MR group, while rocuronium 0.3 mg/kg is given to the MR group. The dose of rocuronium (ED95) is determined based on previous studies which suggest that this dose is sufficient for intubation of children under sevoflurane induction [12, 13]. After 2 min, one of two experienced, board-certified anesthesiologists (EK and SHB) will insert a disposable flexible LMA (LM-FLS, Tuoren Medical Device Co., Ltd., Henan, China) using a standard insertion technique [6]. The LMA is fully deflated and lubricated with a water-based jelly on its posterior surface before insertion. The LMA is pressed along the palate-pharyngeal curve using the index finger, which is positioned at the junction of the airway tube and the LMA cuff. Once the LMA begins to descend into the posterior oropharynx, the non-dominant hand pushes the LMA further down until resistance is felt. The cuff is then inflated until the cuff pressure reaches 40 cmH_2_O according to a manometer (VBM, Medizintechnik, Sulz, Germany), and the required inflation volume is recorded. The selection of LMA size is made according to the manufacturer’s directions. Successful insertion is determined by observation of the square-wave tracing on the capnography, thoracoabdominal movement, bilateral auscultation, and a sealing pressure > 15 cmH_2_O [8].

Anesthesia is maintained with 1.5–2.0 minimum alveolar concentration (MAC) of sevoflurane and 50% oxygen, with a BIS target range of 40–60. No nitrous oxide is used. At the end of the surgery, inhalation agents are discontinued, and 100% oxygen at 6 L/min is administered. Pyridostigmine and glycopyrrolate mixtures are administered to antagonize the neuromuscular blockade in the MR group, whereas the same dose of saline is administered in the Saline group. All patients are given prophylactic antiemetics with dexamethasone 0.2 mg/kg and ondansetron 0.1 mg/kg. The LMA is removed after the patient begins to breathe regularly. Prior to removal, the LMA cuff is deflated, and any oral secretions are gently suctioned. Careful observation, with supplemental 100% oxygen through a facial mask, is carried out for approximately 5 min after the LMA removal. During this period, an attending anesthesiologist needs to be prepared for desaturation or apnea. The patient is then delivered to the PACU and kept under close observation by the anesthesiologist.

### Measurements

The primary outcome measurement is the oropharyngeal leak pressure (OLP) at a fixed gas flow of 3 L/min just before the start of surgery. Secondary outcomes are intubation time for successful insertion, ease of insertion and mask bagging, intubation attempts for successful insertion, successful insertion rate on the first attempt, hemodynamic parameter changes before and after the insertion, ventilator parameter changes such as peak inspiratory pressure (PIP) and expiratory tidal volume to set tidal volume ratio (TVe/TVs) before and at the end of surgery, the fiberoptic view of the LMA position after the successful insertion, as well as postoperative complications. Other outcomes include extubation time, eye opening time, Watcha agitation score, the Face, Legs, Activity, Cry, and Consolability (FLACC) score during the PACU stay, the amount of analgesics administered during the PACU stay, and duration of the PACU stay.

The modified Yale Preoperative Anxiety Scale (mYPAS) is checked when the patients arrive at the preoperative waiting room with their parents before the premedication [14]. The OLP is determined by closing the expiratory valve at a fixed gas flow and then recording the airway pressure indicated by the anesthesia machine at which an audible gas leak is detected in the mouth [15]. Intubation time for successful insertion is measured from the time of opening the mouth until the time of bilateral chest auscultation and detection of capnography. The insertion attempt is stopped and mask bagging is applied when oxygen saturation falls below 90%. Failure of insertion is defined as the unsuccessful insertion of the device after three attempts. In this case, 0.3 mg/kg rescue rocuronium is administered, and another attempt to insert the LMA is made after 2 min. If an additional attempt is not possible, we will intubate the tracheal tube using direct laryngoscopy. Ease of insertion and mask bagging is subjectively graded by the investigator him/herself as easy, moderate, or difficult. Hemodynamic parameters such as the mean blood pressure and heart rate are recorded before and after the insertion. After fixation of the LMA by taping the tube over the chin, a 3.1-mm fiberoptic bronchoscope (PortalView® LF-DP; Olympus Medical Systems Co., Tokyo, Japan) is introduced just proximal to the end of the ventilator conduit, and the laryngeal view is evaluated according to the method described by Timmermann and colleagues [16]. An optimal view is defined as follows: the tip of the LMA placed behind the arytenoids, the epiglottis is visible, not folded down and not covering the airway, and the vocal cords are completely visible under the epiglottis. Any condition present that falls outside of these criteria is judged as a suboptimal position. At the end of the surgery, end-tidal sevoflurane concentration (ET sevo%), BIS, and train of four (TOF) ratio are recorded. Extubation time is defined as the time from the end of the surgery until the time of removal of the device.

Upon admission to the PACU, agitation and pain scores are measured every 10 min until discharge. Agitation is measured by the Watcha scale, which consists of four possible scores: 1, calm; 2, crying but can be consoled; 3, crying and cannot be consoled; 4, agitated and thrashing around [17]. Pain is measured by FLACC scores ranging from 0 to 15 [18]. If the Watcha score is > 2, FLACC score is > 4, or the patient wants analgesics, fentanyl 0.5 mcg/kg will be administered. This dose can be repeated if the symptoms do not subside, or ketorolac tromethamine 0.5 mg/kg can be administered.

Postoperative complications such as respiratory adverse events (coughing, laryngospasm, bronchospasm, postoperative stridor, and desaturation; SpO_2_ < 95%) [19], gastric insufflation, excessive secretion, postoperative nausea and vomiting, sore throat, and bloody tinge on the LMA surface are recorded. Coughing is defined as persistent coughing lasting more than 10 s. The severity of sore throat is classified using a four-point scale as follows: none, no sore throat; mild, complained of sore throat only upon inquiry; moderate, complained of sore throat without inquiry; severe, change of voice or hoarseness associated with throat pain [20].

The patients are discharged when they are calm and meet a modified Aldrete score ≥ 9 [21], and the duration of the PACU stay is recorded as the PACU stay time.

### Sample size

The primary outcome is the OLP. Just as in our study, a previous study confirmed the successful insertion of LMA as a sealing pressure (OLP) > 15 cmH_2_O [8]. We thought that a risk difference of 10% of this minimal sealing pressure would be appropriate to show the non-inferiority of non-MR group over MR group based on our clinical judgement. Therefore, we set the non-inferiority margin to 1.5. A sample size of 58 in each group is needed to achieve 80% power with an alpha of 0.025 to detect non-inferiority. The data are drawn from our pilot study with standard deviations (SDs) of 3.0 and 2.7. Anticipating a 10% dropout rate, we plan to enroll a total of 64 patients per group. The sample size is calculated using PASS software (version 15.0, NCSS, Kaysville, UT, USA).

### Statistical analysis

Both intention-to-treat and per-protocol analyses will be performed. After testing the normality assumption using the Kolmogorov-Smirnov test, continuous variables will be expressed as the mean and the SD or the median and the interquartile range (IQR). Categorical variables will be expressed as numbers of patients and proportions.

The primary outcome (OLP) will be compared with a non-inferiority analysis. The non-inferiority of the LMA insertion without muscle relaxants (T, test) over the LMA insertion with muscle relaxant (C, active comparator) will be accepted if the upper bound of the two-sided 95% confidence interval for C-T is less than the assumed non-inferiority margin of 1.5. For secondary outcomes, continuous variables will be examined with an independent samples *t* test or the Mann-Whitney *U* test, and categorical variables will be examined with Pearson’s chi-squared test or Fisher’s exact test. All tests are two-sided, and *P* < 0.05 is considered statistically significant. A statistician who was not involved in data collection will conduct all statistical analyses using SPSS software (version 19.0, SPSS Inc., IBM, Chicago, IL, USA).

## Discussion

Repeated and prolonged attempts of LMA insertion or reposition of a misplaced LMA may contribute to adverse respiratory events such as laryngospasm, hypoxemia, and pharyngeal mucosal injury. To improve the clinical performance of LMA insertion or to achieve optimal positioning, many factors affecting the LMA insertion should be evaluated and optimized. Of these, muscle relaxants have long been used with a belief that they can positively influence upper airway anatomy and ventilation efficacy. Contrary to this belief, it was reported that ventilation efficacy (represented by tidal volume and airway flow) and pharyngeal mucosal pressure were not altered after rocuronium administration [22, 23]. The increase in airway dilation after succinylcholine administration was temporary during initial pharyngeal fasciculation [22]. Nevertheless, some studies reported that muscle relaxants facilitated LMA insertion in anesthetized adult patients [8–10]. Since the pediatric airway differs anatomically from the airway of adults [4–7], LMA insertion in children can also differ from that in adults. Therefore, we designed this prospective, randomized, double-blind trial to evaluate the efficacy of muscle relaxants during the insertion of LMA in pediatric patients.

The sealing pressure of the LMA, or the OLP, is a marker of the pressure exerted by the cuff of the LMA against the pharyngeal mucosa. To avoid the risk of gastric insufflation and consequent gastric regurgitation, higher sealing pressure is the cornerstone of LMA use. In a previous study, a positive correlation was found between the OLP and the directly measured mucosal pressure [24]. Therefore, we thought that if muscle relaxants change the pharyngeal structures, they may also affect the OLP. Indeed, various studies have measured the OLP as a primary outcome to evaluate the efficacy of LMA insertion [25–27]. We also measure ease of insertion, insertion time for successful insertion, ventilator parameters, and fiberoptic bronchoscopy (FOB) view of the LMA to judge the quality of LMA insertion with or without muscle relaxants. To compare the residual effects of muscle paralysis and airway-related complications using muscle relaxants, we also check for postoperative respiratory adverse events such as desaturation, cough, stridor, laryngospasm, bronchospasm, and the degree of sore throat.

In pediatric anesthesia, the need for muscle relaxants has decreased with the development of new anesthetic agents and the pursuit of minimally invasive surgeries [13, 28]. If an adequate depth of anesthesia is maintained to suppress airway reflexes, movement, and hemodynamic response and to keep appropriate surgical conditions, an LMA can be maintained throughout an operation without administration of a muscle relaxant. Hence, we questioned whether muscle relaxants have beneficial effects on the clinical performance of LMA manipulation. To our knowledge, there is a lack of studies that investigate whether muscle relaxants yield any advantages on the clinical performance of LMA insertion and placement in pediatric patients under general anesthesia. If successful, this study will provide a resource to improve the process and safety of using pediatric LMAs under general anesthesia.

### Trial status

The recruitment commenced in April 2018 and aims to enroll 128 participants for the trial. It is anticipated that recruitment will end by April 2019.

## Additional file

Additional file 1: SPIRIT checklist. (DOC 123 kb)

## Abbreviations

BIS: Bispectral index
ET sevo %: End-tidal sevoflurane concentration
FLACC: Face, Legs, Activity, Cry, and Consolability
IQR: Interquartile range
IRB: Institutional Review Board
LMA: Laryngeal mask airway
mYPAS: Modified Yale Preoperative Anxiety Scale
OLP: Oropharyngeal leak pressure
PACU: Post-anesthesia care unit
PIP: Peak inspiratory pressure
TOF: Train of four
